# Occurence of microplastics in the hyporheic zone of rivers

**DOI:** 10.1038/s41598-019-51741-5

**Published:** 2019-10-24

**Authors:** S. Frei, S. Piehl, B. S. Gilfedder, M. G. J. Löder, J. Krutzke, L. Wilhelm, C. Laforsch

**Affiliations:** 1 Department of Hydrology, Bayreuth Center of Ecology and Environmental Research (BAYCEER), University of Bayreuth, Bayreuth, Germany; 2 Limnological Research Station, Bayreuth Center of Ecology and Environmental Research (BAYCEER), University of Bayreuth, Bayreuth, Germany; 3 Department of Animal Ecology I, Bayreuth Center of Ecology and Environmental Research (BAYCEER), University of Bayreuth, Bayreuth, Germany

**Keywords:** Freshwater ecology, Hydrology

## Abstract

Although recent studies indicate that fluvial systems can be accumulation areas for microplastics (MPs), the common perception still treats rivers and streams primarily as pure transport vectors for MPs. In this study we investigate the occurrence of MPs in a yet unnoticed but essential compartment of fluvial ecosystems - the hyporheic zone (HZ). Larger MP particles (500–5,000 µm) were detected using attenuated total reflectance (ATR) - Fourier-transform infrared (FTIR) spectroscopy. Our analysis of MPs (500–5,000 µm) in five freeze cores extracted for the Roter Main River sediments (Germany) showed that MPs were detectable down to a depth of 0.6 m below the streambed in low abundances (≪1 particle per kg dry weight). Additionally, one core was analyzed as an example for smaller MPs (20–500 µm) with focal plane array (FPA)- based µFTIR spectroscopy. Highest MP abundances (~30,000 particles per kg dry weight) were measured for pore scale particles (20–50 µm). The detected high abundances indicate that the HZ can be a significant accumulation area for pore scale MPs (20–50 µm), a size fraction that yet is not considered in literature. As the HZ is known as an important habitat for invertebrates representing the base of riverine food webs, aquatic food webs can potentially be threatened by the presence of MPs in the HZ. Hyporheic exchange is discussed as a potential mechanism leading to a transfer of pore scale MPs from surface flow into streambed sediments and as a potential vector for small MPs to enter the local aquifer. MPs in the HZ therefore may be a potential risk for drinking water supplies, particularly during drinking water production via river bank filtration.

## Introduction

Rivers and streams represent a primary input vector for microplastics (MPs) into marine ecosystems^1,2^. In contrast to marine environments, still little is known about the fate and behavior of MPs in fluvial ecosystems^1^. Only recently MP research has begun to shift its focus from a marine-centric viewpoint towards freshwater and terrestrial systems. MP particles enter fluvial systems from e.g. waste water treatment plant (WWTP) effluents^3^, sewer overflows during heavy rain events, agricultural runoff, aerial input/atmospheric fallout^4,5^, road runoff or via fragmentation of plastic litter^6–8^. WWTPs alone are estimated to contribute up to 520,000 tons per year of MPs to rivers and streams in Europe^6^.

The hyporheic zone (HZ) is an ecologically essential and sensitive compartment of fluvial ecosystems and is defined as the area below the streambed interface that is equally influenced by surface and groundwater flow dynamics^9^. The HZ is known as an important habitat for various invertebrates, being representative for the lower level of the riverine food web and as an important fish spawning area^10^. Although not investigated up to now, compared to MPs that are transported in the open channel flow, MPs located in the HZ should face a much longer retention and thus exposure time to benthic organisms. Although clear evidence is still missing, it is possible that MPs in the HZ are taken up by benthic organisms and are transferred to higher trophic levels in the food web. Thus, the HZ potentially functions as an additional entry point of MPs into riverine food webs. An uptake of MPs in freshwater organisms of different feeding guilds has already been demonstrated^11^, however, data on the effects of MPs on freshwater species is scarce^12^. MPs are known to contain a multitude of chemical additives such as flame retardants or plasticizers that are often carcinogenic or hormone-active, exhibiting a high potential for leaching^13^ and accumulation in higher trophic levels^12^. In addition, environmental contaminants of concern (e.g. heavy metals or pesticides) may adsorb to the surface of the particles^14–16^ and can be transferred to the respective organisms after ingestion^17^. However, the environmental relevance of MPs as a vector for harmful substances is still discussed controversially in the scientific community^16,18^. Furthermore, pathogens or harmful microorganisms may be a component of the biofilm covering MP particles^19–21^ especially after transit through WWTPs.

Until now only few studies have addressed the accumulation of MPs in streambed sediments (Table 1). For the Rhine/Main catchment Klein *et al*.^22^ investigated MP contamination of stream sediments at the confluence between the Main and the Rhine Rivers. MP abundances ranged from 228–3,763 particles per kg dry weight (in the latter referred to as particles/kg) for the Rhine and 786–1,368 particles/kg for the Main River sediments. In the river Thames (UK) average particle abundances in the sediments ranged from 185–660^23^ particles/kg, similar values (on average 760 particles/kg) were measured for lake Ontario tributaries^24^ (Canada) and for the Bijiang River^25^ (178–554 particles/kg) in China. With 75,000 particles/kg^26^, very high MP abundances were measured at contamination hot spots in the Mersey/Irwell River catchment (UK). All these studies show that streambed sediments can contain a much higher contamination than river surface waters and that streambed sediments are significant accumulation areas for MPs in fluvial ecosystems^4^.

**Table 1:** Measured MP abundances in streambed sediments for different fluvial systems.

River	Particle abundance (dry weight) [kg^−1^]	Detected particle size [μm]	Reference
Rhine River (Germany)	228–3,763	63–5,000	Klein *et al*.^22^
Main River (Germany)	786–1,368	63–5,000	Klein *et al*.^22^
Lake Ontario tributaries (Canada)	average 760 max: 28,000	250–5,000	Ballent *et al*.^24^
River Thames (UK)	185–660	1,000–4,000	Horton *et al*.^23^
Beijiang River (China)	178–554	?-5,000	Wang *et al*.^25^
Mersey/Irwell River (UK)	300–75,000	63–5,000	Hurley *et al*.^43^

In the studies listed in Table 1 MP particles <50 µm were not included, most likely due to the high effort necessary to detect this particle size fraction. Particles with an effective size of 50 µm and below can be classified as pore scale and sub-pore scale MPs. This upper limit roughly represents the maximum in the pore size distribution of a medium-grained sand^27^. Evidence for the presence of pore scale MPs in streambed sediments currently is missing in literature. Mainly because the extraction, purification and detection of this size fraction in natural sediments is extremely time and labor intensive and requires spectroscopic techniques for producing reliable data. Ingestion of MP particle sizes at the pore scale by organisms living in the HZ, may involve the potential biological risk of MPs translocation into organs^28–30^ and tissues^26,28,31^ leading to a risk for bioaccumulation, as shown for marine and freshwater food webs^32–35^. Thus data on MP contamination of the HZ are necessary to assess if it represents a potential environmental risk for benthic organisms as well as for freshwater food webs.

**Figure 1:**
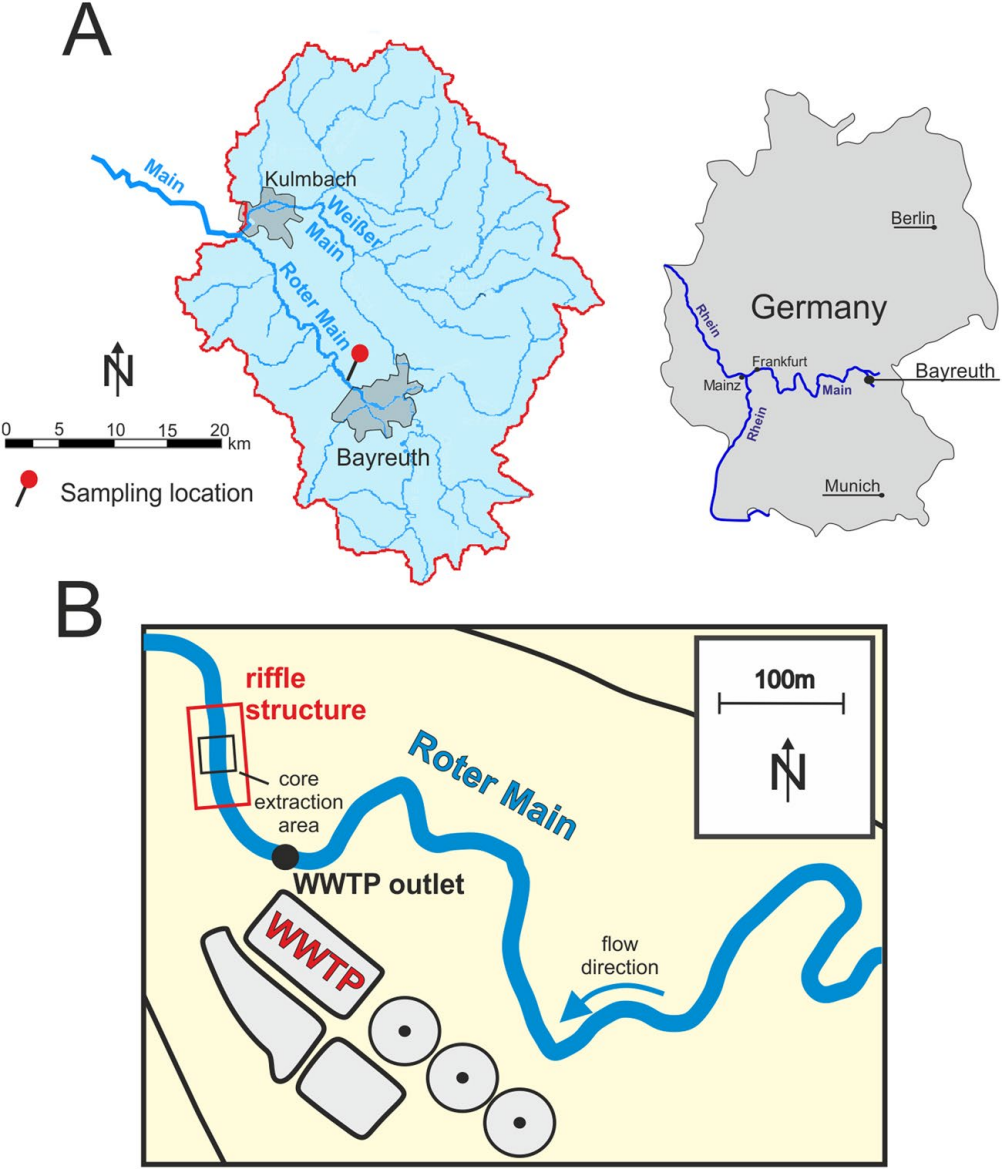
(**A**) Catchment of the Main River tributaries Roter Main River and Weißer Main River. (**B**) Location of the sampling site close to the WWTP north of Bayreuth. All five freeze cores were extracted within an area of 5 × 5 meters.

To contribute towards a better and improved understanding of the fate and behavior of MPs in fluvial ecosystems and to account for the missing data on pore scale MPs, we investigated the depth specific occurrence of MP particles in-between a size of 20–5,000 µm. We extracted five freeze cores from the Roter Main River sediments (southeast Germany), taken from a 5 m × 5 m riffle area located downstream of a WWTP (Fig. 1). To investigate the depth specific MP contamination of the HZ, the cores were sliced into different depth segments. After further sample preparation using sequential filtration the analysis of the larger MPs (500–5,000 µm) was performed with state-of-the-art ATR-FTIR spectroscopy. One core was analyzed as an example for smaller particles (20–500 µm) including pore scale MPs (20–50 µm) with µFTIR spectroscopy after enzymatic purification and extraction. Although we did not perform a detailed MP examination of the Roter Main River sediments over time, our results - for the first time – (1) show depth specific data on the occurrence of MPs in the HZ and (2) further provide first insights in the depth-specific distribution of MP polymer types and size fractions in streambed sediments and the HZ.

## Results

### MPs 500–5,000 µm

**Figure 2:**
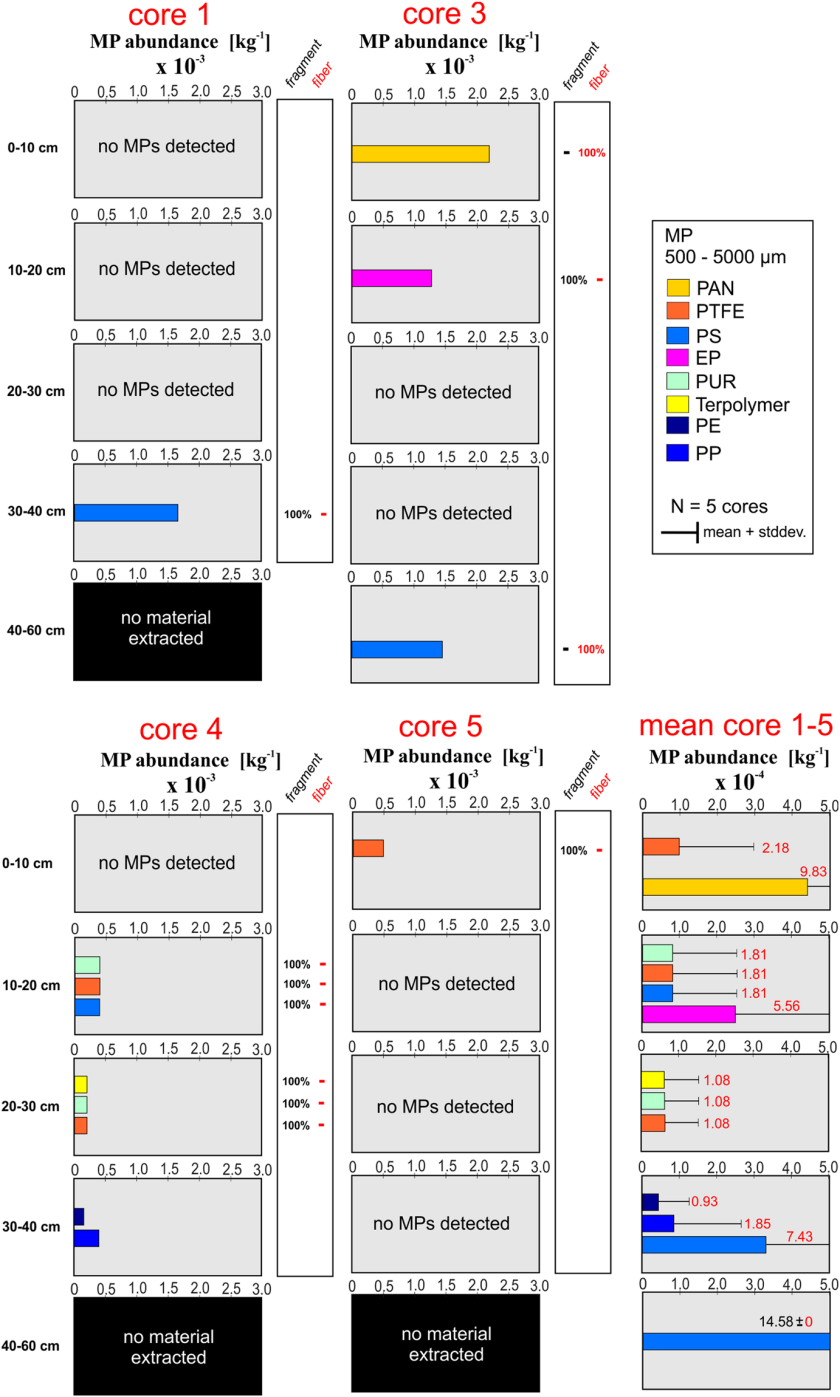
Depth specific distribution of MP particles 500–5,000 µm (per kg dry weight), extracted from the five freeze cores of the river Roter Main River (PAN = polyacrylonitrile, PTFE = polytetrafluoroethylene, PS = polystyrene, EP = epoxide, PUR = polyurethane, PE = polyethylene, PP = polypropylene). Corresponding dry weights of the sub-samples are presented in the Supplementary Material. The red numbers inside the bar plots represent the estimated standard deviation. Results for core 2 are not shown as no MPs (500–5,000 µm) were detected for this core.

MP particles >500 µm were detected in four of the five extracted cores down to a maximum depth of 60 cm below the streambed interface. All five cores were extracted from a 5 × 5 m area of the riffle structure located close to the outlet of the nearby WWTP (Fig. 1). Extracted material for all five cores in general consisted of coarse sediments with a high fraction of sand, medium to fine gravels and cobbles (classification system after Buffington and Montgomery^36^). For core 1, 2, 4 and 5, no sediment material could be extracted for the deepest core segment (40–60 cm), due to the coarse material present there. All cores were analyzed for MPs > 500 µm by using ATR-FTIR spectroscopy. The verified particle number was normalized to the dry weight of the different segment samples (Fig. 2). Detected MP abundances for the five cores ranged from 0 to 2.2 × 10^−3^ particles/kg. We found no clear pattern in MP contamination as particles in the analyzed cores were non-uniformly distributed with respect to (1) depth specific abundances, (2) polymer compositions and (3) particle shapes (Fig. 2). For several depth segments of the cores no MP particles were found and core 2 was entirely free from MPs > 500 µm.

Based on the extrapolated depth specific abundances obtained from ATR-FTIR spectroscopy a mean value and standard deviation could be estimated (Fig. 2) for the different depth classes. As we only were able to extract one single replicate for the 40–60 cm segment from core 3, a corresponding mean and standard deviation for this segment could not be estimated. The low mean values for MP abundances, ranging from 0.5 × 10^−4^ to 4.4 × 10^−4^ particles/kg, in combination with the high standard deviations reflect the non-uniform MP accumulation in the five cores for particles >500 µm. Plastic polymers detected for MPs > 500 µm in the core segments were polyacrylonitrile (PAN, mean: 9.83 × 10^−4^ particles/kg), polystyrene (PS, mean: 1.81–7.43 × 10^−4^ particles/kg), epoxide (EP, mean: 5.56 × 10^−4^ particles/kg), polytetrafluoroethylene (PTFE, mean: 1.08–2.18 × 10^−4^ particles/kg), polypropylene (PP, mean: 1.85 × 10^−4^ particles/kg), polyurethane (PUR, mean 1.08–1.81 × 10^−4^ particles/kg), terpolymer (mean: 1.08 × 10^−4^ particles/kg), and polyethylene (PE, mean: 0.93 × 10^−4^ particles/kg) (Fig. 2). Expect for PE and PP all detected polymers are non-buoyant. Most of the detected particles >500 µm were irregularly shaped fragments and to a lesser extent fibers (Fig. 2). Fibers made of PAN and PS were exclusively found in core 3 for the shallowest (0–10 cm) and deepest segment (40–60 cm), respectively.

### Size fraction 20–500 µm

**Figure 3:**
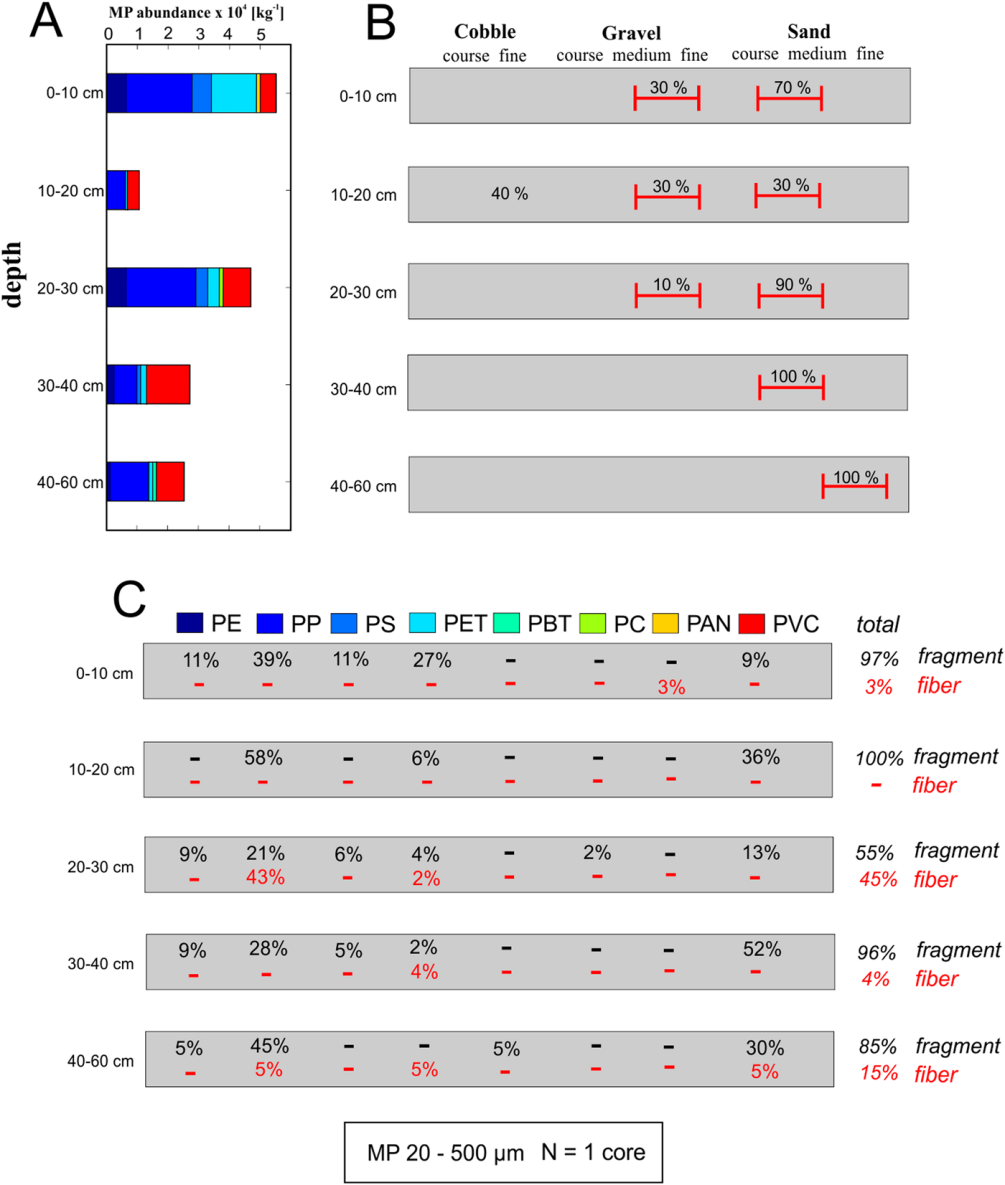
(**A**) Depth specific abundances (per kg dry weight) for MP particles (20–500 µm) in one sediment core of the Roter Main River. Corresponding dry weights of the sub-samples are presented in the Supplementary Material (for color code see C). (**B**) Textural properties of the sediments extracted with the freeze core technique, classification according to Buffington and Montgomery^36^ for hyporheic sediments. (**C**) Depth specific material (PE = polyethylene, PP = polypropylene, PS = polystyrene, PET = polyethylene therephthalat, PBT = polybutylene terephthalate, PC = polycarbonate, PAN = polyacrylonitrile, PVC = polyvinyl chloride) and shape composition for the detected particles. Figures 3 and 4 present data analyzed from the same freeze core.

**Figure 4:**
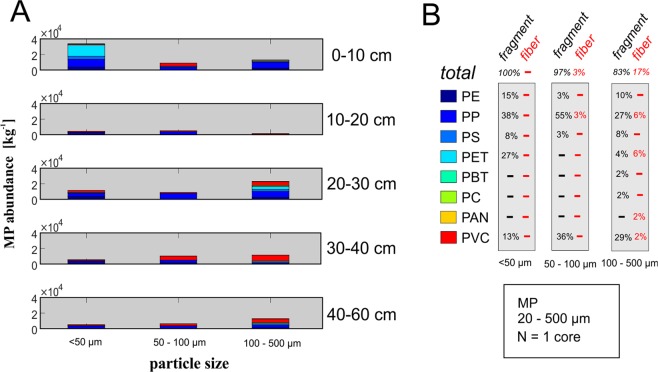
(**A**) MPs-abundances (per kg dry weight) for the different particle size fraction of the sediment core was analyzed down to 20 µm. Corresponding dry weights of the sub-samples are presented in the Supplementary Material. Particles below 50 µm are considered as pore scale and sub-pore scale MPs (for color code see **B**). (**B**) Material and shape composition for the different particle size fractions. Figures 3 and 4 present data obtained from the same freeze core. MP abundances were extrapolated to the dry weights of the samples. (PE = polyethylene, PP = polypropylene, PS = polystyrene, PET = polyethylene therephthalat, PBT = polybutylene terephthalate, PC = polycarbonate, PAN = polyacrylonitrile, PVC = polyvinyl chloride).

As an example for pore scale MPs, core 3 was analyzed for MPs in the range of 20–500 µm using FPA-based µFTIR spectroscopy. The depth specific distribution of MPs in this size range is shown in Figs 3 and 4. A classification of the extracted streambed materials for core 3 is provided in Fig. 3B, according to the classification system proposed by Buffington and Montgomery^36^. In general, MP accumulation decreased with increasing depth. Highest MP accumulation for particles in-between 20–500 µm was found for the upper 10 cm of the core (exceeding 50,000 particles/kg) while lowest particle abundance (~10,000 particles/kg) was detected for the 10–20 cm segment. High MP abundances, ranging from 4,500–30,000 particles/kg, were detected for pore scale MPs (20–50 µm) in the different core segments (Fig. 4A). With ~30,000 particles/kg pore scale MPs especially dominated the superficial core segment (0–10 cm) (Fig. 4A). The size class 100–500 µm displayed the highest abundance in the core segments 20–30 cm and 40–60 cm. In the analyzed core, with the exception of the 10–20 cm segment, there seems to be a tendency towards a lower accumulation of MPs with increasing depth.

The most abundant plastic polymers found in the size range of 20–500 µm particles were PP, polyvinyl chloride (PVC) and polyethylene terephthalate (PET). Non-buoyant polymer types represented a major fraction of detected particles and were found in every depth segment. An exceptionally high number of PP fibers (45%) was found in-between 20 and 30 cm below the streambed interface (Fig. 3C). With increasing particle size, the percentage of fibers increases from 0% for the 20–50 µm class to 3% for the 50–100 µm class and 16% for the 100–500 µm class (Fig. 4B). Majority of the pore scale MPs (20–50 µm) detected for the upper 10 cm of the core were identified as PET particles (~15,000 particles/kg) (Fig. 4A). Other polymer types, beside PET, identified for pore scale MPs (20–50 µm) were PP, and to a lesser extent PVC, PS and PE. All detected pore scale particles were fragments (Fig. 4B). For MPs of the size fraction 50–100 µm and 100–500 µm, PVC and PP were the most abundant polymers that could be verified for almost every depth segment of the analyzed core. Additionally to MP particles <500 µm, some fibers/particles >500 µm were found in the samples, which were excluded as potential contamination and thus not considered further.

## Discussion and Implications

**Figure 5:**
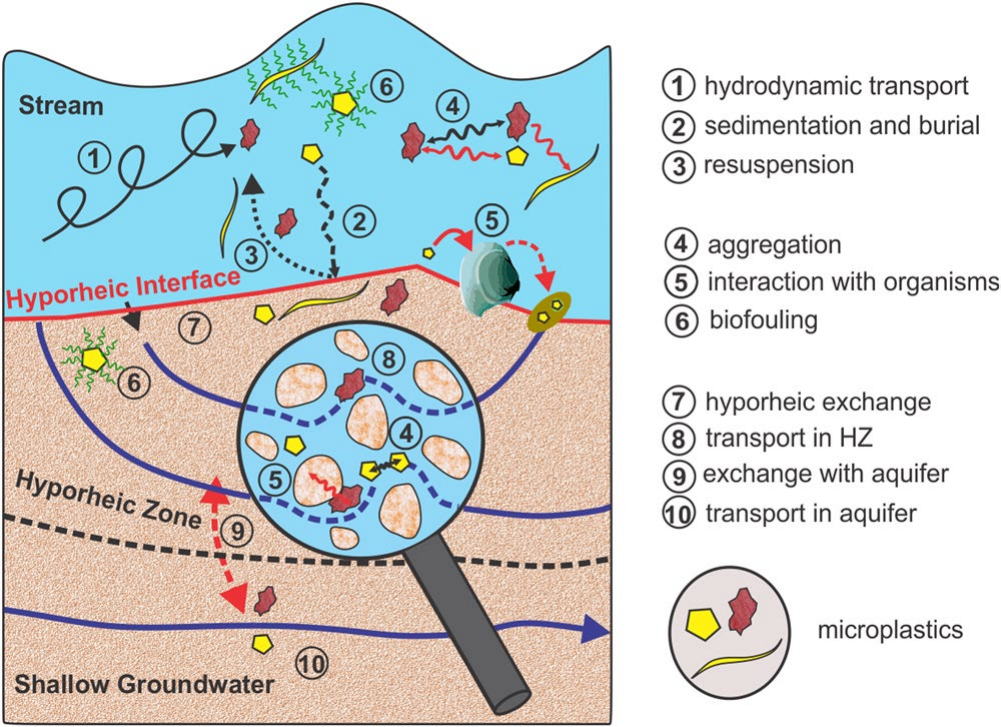
Potential processes that control the transport and redistribution of microplastic particles in fluvial systems.

In fluvial systems, MPs - similar to natural particles - are not uniformly transported. Mechanisms and timescales of transport depend on the hydrodynamic properties of the MP particles such as particle size, shape, density, surface roughness, and the hydrodynamic transport conditions in the open channel flow. Currently there is only a rudimentary understanding of the relevant mechanisms that control the transfer of MPs from surface flow into streambed sediments. Settling of suspended MPs due to the influence of gravity (sedimentation) seems to be the most obvious mechanism leading to a translocation of MPs from the stream into the HZ (Fig. 5). Sedimentation can also affect buoyant MPs after density modification processes occurring in aquatic environments leading to higher gross densities of the respective particles. Such density modification processes include aggregation with other MP particles, sediments or organic matter^37,38^, photo-degradation and biofouling^39^, ingestion and excretion of MPs in fecal pellets by organisms^37^ (Fig. 5).

Small particles such as pore scale MPs are more affected by aggregation processes, as the surface to volume ratio and thus the associated surface reactivity is much higher compared to larger particles^40^. For buoyant synthetic polymers detected in the sediments of the Roter Main River (PE and PP) density modification processes can explain the high abundancies in the HZ. Although abundances for PP and PE particles <500 µm are highest for the upper 10 cm of the extracted core, both lightweight polymers were found in almost every depth segment. For the core analyzed for MPs < 500 µm, including pore scale particles (20–50 µm), buoyant polymers represent a major fraction (depth averaged fractions: 61% for 20–50 µm particles, 63% for 50–100 µm particles and 35% for 100–500 µm particles) of the detected particles and additionally were found among larger MP particles >500 µm (depth averaged fractions: 20%).

In the sediments of the Roter Main River particles >500 μm were found in 4 of the 5 freeze cores and were non-uniformly distributed for the different depth segments. An advective transfer of particles, across the streambed interface due to hyporheic exchange (Fig. 5), can be excluded for MPs exceeding the average pore size of the streambed material (>50 μm). Beside interactions with organisms, sedimentation and burial seems to be the only plausible transfer mechanism for MP particles >50 μm that has led to an accumulation over time in the sediments of the Roter Main River. Hydrodynamic conditions, controlling the transport and sedimentation behavior of natural particles and MP particles alike, in rivers and streams are non-stationary and can vary spatially on very small scales such as a few centimeters^41^. Temporal and spatial variations in transport and sedimentation patterns along the streambed interface can explain the non-uniform distribution of MP particles >500 µm observed for the different cores. Even for the small sampling area (5 m × 5 m) for which the cores were extracted, conditions for sedimentation of MPs were non-uniform and affected by the dynamical variations of the streambed interface and the hydrodynamic characteristics of open channel flow. As the depth specific sediment composition of the core segments indicate previous sedimentation conditions in the stream, the sporadic presence of MPs > 500 µm in the core likely also reflects these non-stationary sedimentation characteristics. Here the different polymer types (MPs > 500 µm) detected in the cores likely were affected differently in the past regarding their transport and accumulation behavior before the particles where immobilized in the HZ.

Once in the streambed sediments larger MPs are temporarily immobilized likely resulting in a higher exposure time to benthic organisms increasing the probability for uptake. Uptake and excretion of MPs by benthic organisms can reverse the extent of biofouling of MP particles by digesting biofilms in which case polymers like PP and PE may regain their buoyancy^42^. Re-mobilization of MP in streambed sediments can occur during high flow events where high flow conditions lead to streambed erosion (Fig. 5). Such high flow events can have a dramatic effect on MP contamination in streambed sediments, as has been shown recently for the Mersey and Irwell Rivers, where around 70% of the MP loads stored in the riverbed were flushed during a single high flow event^43^. For larger MPs > 500 µm it was reported that those particles were found within the cases of benthic organisms such as the caddisfly larva^44^. Binding of sediments with silk strands from the caddisfly can have a stabilizing effect on the surrounding sediments^45^ providing a mechanism where MPs are retained in the sediments during high flow conditions^44^.

Based on the results obtained for the single core analyzed as an example for MPs < 500 µm, extrapolated abundances increase considerably with decreasing particle size. Highest abundance was estimated for pore scale particles (~30,000 particles/kg) for the superficial segment of the analyzed core. Although clear evidence is still missing, the hypothesis that MP particles at the pore and sub-pore scale can be transferred advectivley via hyporheic exchange (Fig. 5) into the streambed sediments is suggested. For the streambed of the Roter Main River we hypothesize that the accumulation of MPs is controlled by a combination of (1) sedimentation and (2) advective transfer of pore scale particles across the streambed interface. In this context, the observed depth specific accumulation of MPs, where both pore scale and larger particles are equally detectable in high numbers, can be interpreted as the result of both accumulation mechanisms. Hyporheic exchange of water between the stream and the HZ -and likely also of pore scale MPs- is controlled by site specific characteristics such as the morphological shape of the streambed^9^, the influence of local groundwater^46,47^, material heterogeneities in the HZ^48^ and the turbulent conditions in the stream^47^. Pore scale MPs can further decay into nano-sized particles^49^, and for these particles an advective transfer across the hyporheic interface is even more likely. For pore scale MP particles capable of entering the pore system of the HZ, hyporheic exchange represents an additional transfer mechanism that has not yet been recognized.

As part of this study we only analyzed five different freeze cores to investigate the depth specific occurrence of MPs of different size ranges and polymer compositions in the HZ of the Roter Main River. Each of the cores was sliced into five different depth segments and respectively analyzed for MPs. Results of detected MPs were normalized to kg dry weight. For the single core, which was analyzed for MPs < 500 µm, only subsamples of the different depth segments were measured by FPA-based µFTIR spectroscopy. Here the standard normalization procedure results in large extrapolation factors resulting in the high presented MP abundances. Thus, findings for MPs < 500 µm from this single freeze core sample only provides a first insight into the depth specific distribution of pore scale MPs in the HZ and cannot be interpreted as an universally valid assumption. However, our data - to our knowledge - are the first one that investigate the depth specific occurrence of MPs and additionally addresses pore size MPs.

Beside its ecological function, the HZ also is an important interface between the stream and the shallow groundwater system. For rivers and streams, the exchange between surface- and groundwater can be highly non-uniform in space and time^50^. Stream sections or entire river reaches can either gain or lose water from or to the local aquifer^50,51^. Under permanent loosing conditions infiltrating stream water first has to pass through the streambed sediments before it reaches the local aquifer. Thus, mobile pore scale MPs in the HZ can then theoretically be transferred advectivley into the aquifer (Fig. 5). That MP particles at the pore scale can principally be mobile in porous environmental media has already been shown for soils^52,53^. Infiltrating river water is commonly used for drinking water supplies^54^ (induced bank filtration technique) and mobile MPs in shallow groundwater therefore can be seen as a potential threat for drinking water production.

WWTPs are widely recognized as potential point sources for the entry of MPs into aquatic environments^3^. Although a fraction of the MPs may be retained in the sewage sludge, a considerable number of particles leave with the cleaned water as only a very limited number of WWTPs possess the ability to filter particles at the end of the cleaning process. As the study site is located close to the headwater area of the Roter Main River, besides the inlet of the WWTP no other point sources for MPs are present in the catchment suggesting that the MPs we found primarily may stem from the nearby WWTP.

Our data clearly indicate a tendency of increasing MP abundances with decreasing particle size. Compared to data obtained further downstream in the Rhein/Main catchment^22^ (>10 particles/kg for the confluence area of the Main River and Rhein River), the sediments of the Roter Main River show a relatively low MP contamination for particles >500 µm (depth averaged: ≪1 particles/kg). Abundances for the Roter Main River sediments however increase severely (>50,000 particles/kg), for particles <500 µm, whereas for the confluence area maximum reported values lie around 1,100 particles/kg for particles in-between 63–500 µm^22^. Highest MP abundance (~30,000 particles/kg) was estimated for pore scale particles (20–50 µm) in the sediments of the Roter Main River. Other studies dealing with MP contamination of streambed sediments did not analyze particles below 63 µm (Table 1), thus a direct comparison with our data is only possible to a limited extent. However, compared to the abundances of the river systems listed in Table 1, total MP abundances exceeding 50,000 particles/kg estimated for the Roter Main River sediments are close to abundances measured at contamination hot spots in the Mersey/Irwell River sediments^43^ (~75,000 particles/kg). Our results indicate that especially pore scale MPs (~30,000 particles/kg) may represent the dominant size fraction in the HZ of the Roter Main River. Based on these findings the assumption that the number of MP particles of the river systems listed in Table 1 would increase significantly if pore scale MPs were additionally considered is likely and intensive research on this issue is imperative. Furthermore, our data show that although we used the best methodology currently available, especially the development of methods that allow for the analysis of a larger sample amounts must be fostered in future research to reduce uncertainties by large extrapolation factors.

## Summary and Conclusions

Our data indicate that the HZ represents an important accumulation zone - and thus a temporal sink - for MPs of various plastic polymer types and particle size fractions in fluvial ecosystems. Besides data on MPs > 500 µm the results obtained from one sediment core serves as an example suggesting that pore scale MPs < 50 μm potentially represent the most abundant size fraction in the sediments of the Roter Main River. As it is highly unlikely that this is only the case for the Roter Main River, we conclude that the importance of MPs of a size at the pore scale and below is given for other river systems as well. We strongly assume that by accounting for MPs < 50 μm, the hitherto presented abundances of MPs in streambed sediments dramatically would increase by orders of magnitudes. We hypothesize that accumulation of MPs in streambed sediments beside sedimentation is additionally controlled by advective transfer of pore scale and sub-pore scale particles across the streambed interface. Under loosing conditions MPs with sizes of or below the pore scale may even reach the shallow groundwater. Up to now freshwater systems have received only limited attention concerning the fate and behavior of MPs. However, it is imperative to address the mechanistic behavior of MPs in future research to better understand the threat MPs poses to fluvial ecosystems and additionally drinking water security.

## Material and Methods

### Study site and freeze core sampling

The Roter Main River is part of the headwater catchment of the Main River. The study site is located close to the city of Bayreuth in southeast Germany (Fig. 1). Previously, the Main River was identified as a major source of MPs for the river Rhine, with WWTPs identified as the major contributor to MP loads^22^. Sampling for MPs in the Roter Main River was performed in May 2016 and August 2017 and involved extracting five freeze cores down to a maximum depth of 60 cm from a natural riffle structure. Freeze core sampling was chosen mainly because the streambed materials involved larger gravels and fragments. For the freeze core extraction, a stainless-steel pipe was hammered into the sediments and filled with a mixture of dry ice and ethanol. The pipe was removed as soon as the surrounding sediments froze on the metal surface of the pipe (~20 minutes). For all cores a cylindrical shaped sediment core was extracted from the riverbed sediments. All five cores where extracted and analyzed for MPs in the size range 500 to 5,000 µm using ATR-FTIR spectroscopy and one core was additionally investigated for MP particles in the size range 20–500 µm which includes pore scale and sub-pore scale particles (20–50 µm) using focal plane array FPA-based µFTIR spectroscopy.

### Sample preparation

The freeze cores were sliced into 10 cm depth segments prior to thawing and sample preparation. For the freeze core that was analyzed for 20–500 µm MPs the last 20 cm had to be combined into a single segment as not enough material was extracted. Each freeze core segment sample was then dried (50 °C) until a constant weight was reached (Sartorius 3802MPS) to allow for a normalization of MP concentrations to kg dry weight. Next, the samples were wetted with filtered de-ionized water and 20 ml of 30% H_2_O_2_ was added. After an incubation time of approximately one hour the samples were sieved sequentially over a 500 and 10 µm stainless steel sieve cascade using filtered de-ionized water. The sample material on the 500 µm mesh was visually inspected under the microscope and every particle that looked like a potential synthetic particle, i.e. had no clear mineral or organic appearance, was sorted out for ATR-FTIR measurement. Those particles were then consequently photographed and transferred into Eppendorf tubes for ATR-FTIR analysis.

In one core sampled in August 2017 the remaining material on the 10 µm mesh was further analyzed for MP particles <500 µm. The sample material was again dried (50 °C) to increase the efficiency of the density separation and a subsample of approximately 50 g (Sartorius LC1201S) of each 10 cm segment was taken. To remove inorganic material from the subsamples a density separation in a separating funnel was conducted with zinc chloride solution with a density of 1.6–1.7 kg/l. After a settling time of 24 hours the heavy sediment material was discarded, and the low-density material including MPs was filtered through a 10 µm stainless steel filter. To remove residual zinc chloride the filter was then rinsed with 98% ethanol and transferred into a laboratory beaker using filtered de-ionized water. Organic material was removed using wet peroxide oxidation (Fenton’s reagent: 0.05 M Fe^2+^ solution and 30% H_2_O_2_)^55^. To allow for an undisturbed spectroscopic analysis^56,57^ a subsample of 1/64 of each 10 cm segment was filtered onto aluminum oxide filters (Anodisc, Whatman) for subsequent FPA-based µFTIR spectroscopy. Subsampling was performed on each final stainless-steel filter via specially designed sample dividing pliers that divides the filter into two equal parts and ensures a maximum of representativeness of the subsamples. One obtained half was then re-suspended in filtered de-ionized water and the filtration onto a stainless-steel filter repeated. This procedure was carried out until 1/64 of each sample was extracted which was subsequently filtered onto aluminum oxide filters for a reliable FPA-based µFTIR measurement.

Measures against contamination included the inclusion of glass and stainless-steel materials wherever possible, filtering of all chemicals and water during sample preparation, constant coverage of samples with aluminum foil or glass lids and the wearing of laboratory coats made of cotton. Possible contamination during laboratory analysis was further monitored using four negative controls, containing filtered de-ionized water only and receiving the same treatment as the samples. The results of the negative controls were subtracted from the sample results and a detailed table including polymer types, forms and size classes of MPs detected within the blanks is provided within Table S2 (Supplementary Material).

### Detection of MPs > 500 µm

Analysis of MPS > 500 µm was conducted using ATR-FTIR spectroscopy^56^ after sample preparation. The FTIR spectra were measured using a Tensor 27 FTIR spectrometer equipped with a Platinum-ATR-unit (Bruker Optics GmbH). The IR spectrum of each potential MP particle was recorded as an average spectrum of 16 co-added scans in the spectral range between 4000–400 cm^−1^ with a spectral resolution of 8 cm^−1^ with the software OPUS 7.5 (Bruker Optik GmbH). The background was measured against air. The sample spectra were compared to reference spectra of a self-made polymer library containing the most common polymers, as well as natural materials and has more than 130 entries^56^. The analyses have been conducted by staff with a long expertise in FTIR analysis. Fouling and other impurities at the particle surfaces may be represented in the spectra, as the ATR-FTIR technique measures the surface of the particles. In such cases the particle surface was cleaned mechanically or by alcohol to receive pure polymer spectra. Only particles that showed clear polymer spectra after cleaning were considered as MPs.

### Detection of MPs < 500 µm

The aluminum oxide filters of the subsamples and negative controls were placed onto a calcium fluoride window in a customized sample holder and measured in transmittance mode with a Hyperion 3000 FTIR microscope (Bruker Optics GmbH) equipped with a 15 × Cassegrain objective and a 64 × 64 FPA detector. All measurements were performed with the settings published in Löder *et al*.^56^ in a spectral range of 3,600–1,250 cm^−1^, with a spectral resolution of 8 cm^−1^ and 6 co-added scans as well as 4 × 4 binning. The analysis of the obtained large, high-resolution chemical imaging datasets was performed with the software ImageLab and a fast and automated analysis via Random Decision Forest Classifiers^58^ for the eleven most important polymers. All selected automatically analyzed particles were again manually checked against the reference database as stated above in cases of unclear spectra.

## Supplementary information

Supplementary Information
