# Supplementary material for: Occurence of microplastics in the hyporheic zone of rivers
Source: Sci Rep. 2019 Oct 24;9:15256. doi: 10.1038/s41598-019-51741-5 (PMC6813303; doi:10.1038/s41598-019-51741-5)

**Occurence of microplastics in the hyporheic zone of rivers.**

Frei S.^1*^, Piehl S.^3^, Gilfedder B.S.^2^, Löder M. G. J.^3^, Krutzke J.^1^, Wilhelm L.^1^, Laforsch C.^3^

^1^Department of Hydrology, Bayreuth Center of Ecology and Environmental Research (BAYCEER), University of Bayreuth, Germany

^2^Limnological Research Station, Bayreuth Center of Ecology and Environmental Research (BAYCEER), University of Bayreuth, Germany

^3^Department of Animal Ecology I, Bayreuth Center of Ecology and Environmental Research (BAYCEER), University of Bayreuth, Germany

*Correspondence to sven.frei@uni-bayreuth.de

Table S1: Dry weights of the freeze core segments and the identified MP-particles

| Freeze Core | Depth Segement | Dry Weight | MPP > 500 μm | MPP < 500 μm detected on Anodisc (FTIR) 50/64 g |
| --- | --- | --- | --- | --- |
| [-] | [cm] | [kg] | [-] | [-] |
|  |  |  |  |  |
| 1 | 10-20 | 2.47 | 3 | not analyzed |
| 1 | 20-30 | 4.11 | 3 | not analyzed |
| 1 | 30-40 | 4.83 | 3 | not analyzed |
| 1 | 40-50 | 2.02 | 0 | not analyzed |
|  |  |  |  |  |
| 2 | 0-10 | 2.05 | 1 | not analyzed |
| 2 | 10-20 | 2.32 | 0 | not analyzed |
|  |  |  |  |  |
| 3 | 10-20 | 2.03 | 0 | not analyzed |
| 3 | 20-30 | 2.28 | 0 | not analyzed |
| 3 | 30-40 | 0.6 | 1 | not analyzed |
|  |  |  |  |  |
| 4 | 0-10 | 1.19 | 0 | not analyzed |
| 4 | 10-20 | 1.5 | 0 | not analyzed |
| 4 | 20-30 | 2.86 | 0 | not analyzed |
|  |  |  |  |  |
| 5 | 0-10 | 0.45 | 1 | 44 |
| 5 | 10-20 | 0.80 | 1 | 10 |
| 5 | 20-30 | 0.5 | 0 | 37 |
| 5 | 30-40 | 0.26 | 0 | 22 |
| 5 | 40-60 | 0.69 | 1 | 20 |

Table S2: Polymer types, forms and size classes of MPs detected within the blanks.


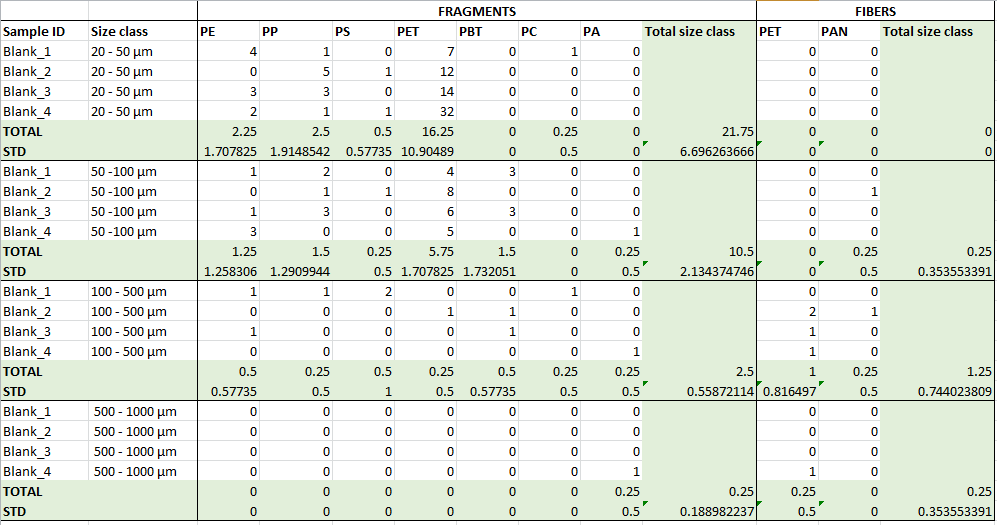

Supplement: Supplementary file 1 — Supplementary Information [file 41598_2019_51741_MOESM1_ESM.docx]
